# The Parenchymatous Rupture Treatment

**Published:** 1895-01

**Authors:** C. Arnold F. Lindorme

**Affiliations:** Atlanta, Ga.


### THE PARENCHYMATOUS RUPTURE TREATMENT.

BY C. ARNOLD F. LINDORME, PH.D., M.D.,
ATLANTA, GA.

The fate which this treatment has met shows again that a practice which is based upon a crooked theory does not secure good results. If the leading thought of a technical procedure is not vested in anatomy and physiology, it cannot become a working theory ; it has no foundation in reality, and if it be ever a success, it is a mere casualty, depending on fortuitous circumstances, the nature of which is unknown and can methodically, therefore, not be taken advantage of.

The first case which came under my observation was an exceedingly captivating one. It was an aged individual and a severe scrotal hernia of long standing. As a rule there was an easy reduction. But repeatedly strangulation had threatened, and when I saw the case the trouble had wrought secondary symptoms, which rendered a remedial interference peremptory.

The injections were made at random. Since there was no fixed theory, the operator was at sea about whether the medicine would work better being thrown in the midst of the ring or into the pillars of the same. But the lack of theory was no impediment of success. After one and a half to two dozen injections, the breach was closed up. Our invalid could lay off his truss, stood and walked more erect, and was complimented on having added a half-score years to his term of life, if no more.

The cure remained by no means unique. But then cases which were not a tenth part so bad proved rebellious, and up to date it remains to the author a sort of mystery why the most desperate of the cases got well so soon, while others, with nearly the same, or a more careful treatment even, were left out and had to be abandoned as incurable.

My own belief in the remedy used was never deep. It did not satisfy me theoretically. Failing, I looked at it all the more skeptically, studied it rationally, and the results attained were rather negative. It read :

℞ Tinct. arnic. hamam ........................................... ℥ ss.

Sulph. zinc ........................................... gr. xxxij.

Aquæ destill ........................................... ℥ j.

M. Filter. Running off clear.

Add alcohol ........................................... ℥ ss.

Inject from eight to twenty-five drops, a dose, as to which I will warn right here ; I never saw any good come from a bigger dose than ten to twelve drops.

Other formulæ given in Henry O. Marcy's book on the radical cure of hernia* I found did not differ much, but were as poor in theory as the above. Sulphate of zinc is an escharotic, hamamelis supposed to be an astringent. How can they work together, and what business has arnica to perform ?

If the idea in the treatment was to set up an inflammation, which the use of the sulphate of zinc would presurmise, then the hamamelis as well as the arnica were misplaced, nor was there any sense in a reiteration of the injection once or twice a week for a lengthy period until cured; every new injection would be a disturbance of the artificial process.

If there was to be a hardening of the fibers of the ring, so as to narrow the slit it offered to a slipping through of intestines, then all the three first items were a very ambiguous addition and the alcohol all by itself the proper remedy to use. I concluded, therefore, to assume the standpoint of the servant girl of a literary celebrity, who superciliously discouraged a book agent soliciting her goodwill with the family by telling him : "No, sir, when *we* want books, we write them ourselves."

By using my own recipe of alcohol all by itself, I obviated, moreover, the concomitant inconvenience with the old remedy of pain and laying up. Injecting no more than ten to twelve minims at most, all the pain caused was restricted to some smarting immediately on the injection, leaving the patient nearly free from all disagreeable sensation afterwards, and owing to this circumstance I could return to it sooner, an injection every other day being none too many.

*Physician's Leisure Library. Ser. III.

Theoretically it was certainly a gain ; since the purpose in using the alcohol is to harden, to draw together, a repetition of the injection is indicated, rational, and sensible. Practically I gained as much perhaps as with the former formula, but had no opportunity to try it on a large enough scale to give definite information, the poor results of the whole treatment invalidating the applications. Rupture treatment is something which can be put off, and times were hard.

There is one point, apparently, which in most cases can be reached. This is a great deal less inconvenience from wearing a truss. The most disagreeable part of the discomfort by the latter is in the consciousness of the afflicted that it let slip the rupture. If now, by the treatment, so much of retention of the intestine is obtained that the truss can be worn with a certain degree of carelessness, this is productive of so much comfort that it well pays for the trouble and the expense of the treatment.

A danger against which those who want to try the treatment must be warned is to throw the medicine, instead of into the outer or the inner ring, according to whether the case is direct or indirect, between the two layers of the superficial fascia. Probably in consequence of the profusion of lymphatics which throng in the latter, the medicine is liable to cause lymphadenitis, which tends to discredit the treatment. The strength in which I used the alcohol was about 70°.

One point more I will mention ere I bring this paper to a close, because I wish others also to make observations. The books say there is one direct hernia to five indirect ones. My experience was the reverse. Among about thirty direct hernias I had only three indirect ones. Is that a peculiarity of Georgia, or have we simply to conclude that there is much obscure, as yet, in the etiology of hernia?

